# Inconsistency analysis between metagenomic next-generation sequencing results of cerebrospinal fluid and clinical diagnosis with suspected central nervous system infection

**DOI:** 10.1186/s12879-022-07729-0

**Published:** 2022-09-30

**Authors:** Jin Wang, Jun Ye, Liqi Yang, Xiangfeng Chen, Haoshu Fang, Zhou Liu, Guomei Xia, Yafei Zhang, Zhenhua Zhang

**Affiliations:** 1 Department of Infectious Diseases, The Second Hospital of Anhui Medical University, Hefei, 230601 China; 2 Department of Infection Management, The Second Hospital of Anhui Medical University, Hefei, China; 3 Department of Pathophysiology, Anhui Medical University, Hefei, China; 4 Department of Clinical Laboratory, The Second Hospital of Anhui Medical University, Hefei, China

**Keywords:** Metagenomic second-generation sequencing, Clinical diagnosis, Inconsistency analysis, Central nervous system infection, Evaluation process

## Abstract

**Background:**

Recently, with the rapid progress of metagenomic next-generation sequencing (mNGS), inconsistency between mNGS results and clinical diagnoses has become more common. There is currently no reasonable explanation for this, and the interpretation of mNGS reports still needs to be standardised.

**Methods:**

A retrospective analysis was conducted on 47 inpatients with suspected central nervous system (CNS) infections, and clinical data were recorded. The final diagnosis was determined by an expert group based on the patient’s clinical manifestation, laboratory examination, and response to treatment. mNGS results were compared with the final diagnosis, and any inconsistencies that occurred were investigated. Finally, the credibility of mNGS results was evaluated using the integral approach, which consists of three parts: typical clinical features, positive results with the traditional method, and cerebrospinal fluid cells ≥ 100 (× 10^6^/L) or protein ≥ 500 mg/L, with one point for each item.

**Results:**

Forty-one patients with suspected CNS infection were assigned to infected (ID, 31/41, 75.61%) and non-infected groups (NID, 10/41, 24.39%) after assessment by a panel of experts according to the composite diagnostic criteria. For mNGS-positive results, 20 of the 24 pathogens were regarded as contaminants when the final score was ≤ 1. The remaining 11 pathogens detected by mNGS were all true positives, which was consistent with the clinical diagnosis when the score was ≥ 2. For mNGS negative results, when the score was ≥ 2, the likelihood of infection may be greater than when the score is ≤ 1.

**Conclusion:**

The integral method is effective for evaluating mNGS results. Regardless of whether the mNGS result was positive or negative, the possibility of infection was greater when the score was ≥ 2. A negative mNGS result does not necessarily indicate that the patient was not clinically infected, and, therefore, clinical features are more important.

**Supplementary Information:**

The online version contains supplementary material available at 10.1186/s12879-022-07729-0.

## Background

Central nervous system (CNS) infections, including meningitis, encephalitis, abscess, and myelitis are caused by various infectious agents. Infectious diseases of the CNS are associated with high morbidity and mortality [1]. In 2016, the World Health Organization estimated 320,000 deaths globally to be related to meningitis [2]. The clinical presentation of CNS infection is non-specific; therefore, clinicians cannot determine the potential causative pathogen based on symptoms and physical examinations and must rely on microbiological tests [3, 4]. Cerebrospinal fluid (CSF) Gram staining and routine biochemical examinations are common diagnostic methods for CNS infections that are not specific to causative pathogens. Bacterial culture and polymerase chain reaction (PCR) tests identify specific pathogens in the CSF and are currently the most important methods for diagnosing CNS infections. However, most pathogens that cause CNS infections cannot be routinely cultured, and most PCR tests are targeted against common pathogens. A specific aetiologic agent cannot be identified in more than 50% of patients with CNS infections [5–7]. The failure to obtain a timely aetiological diagnosis leads to the delay of treatment, which burdens the patient's family and society [2, 8].

Metagenomic next-generation sequencing (mNGS) is a promising method for the diagnosis of infectious diseases. It is a non-priori and non-biased detection method that enables the identification of all potential causes in a single test [9, 10]. Since the detection of the first case of leptospirosis in 2014 [11], mNGS has developed rapidly in the field of infectious diseases and has a significant advantage for the detection of new or rare pathogens. Initial mNGS applications focused on detecting pathogens in sterile specimens, such as CSF and brain biopsies, simplifying the clinical significance of detected microorganisms, improving the detection positive rate of aetiology, and bringing value to the practical clinics [12–14]. However, mNGS has high sensitivity but low specificity compared to microbial culture. Several studies have shown that inconsistency between mNGS results and clinical diagnoses is common [15–17]. Moreover, it is common to detect more than one microorganism in an mNGS result, and the causative pathogen may be one of them or none of them. There are currently no guidelines or studies that provide reasonable explanations for false-negative and false-positive mNGS results or detailed assessment procedures to guide clinicians.

To bridge this knowledge gap, we conducted a retrospective study that collected clinical data from 47 patients with suspected CNS infection between 2018 and 2021. These cases were taken as examples to analyse the causes of false-positive and false-negative results and to explore the rules preliminarily in order to provide a reference for clinicians when analysing mNGS results for suspected CNS infection. Our study aimed to explore the interpretation of mNGS reports in a clinical scenario.

## Materials and methods

### Study design and patient selection

**Fig. 1:**
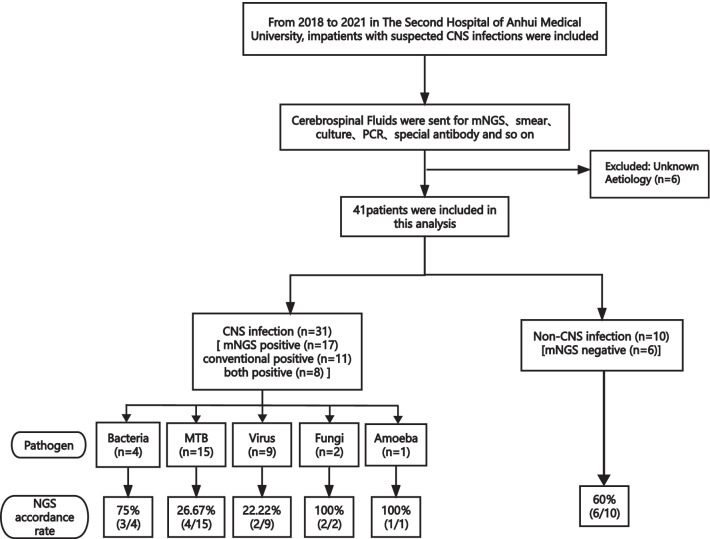
Flowchart of study enrolment process

This retrospective study was conducted on patients with suspected CNS infection who were admitted to the Second Hospital of Anhui Medical University from 2018 to 2021 (Fig. 1). This study was reviewed and approved by the ethics committee of Anhui Medical University [20170216]. Informed consent forms were signed by patients or their surrogates. CSF samples were sent for: routine (quantity and classification of CSF cell) and biochemical (protein, glucose, chloride, and adenosine deaminase content of CSF) tests; culturing of bacteria, fungi and tuberculosis; autoimmune antibody tests; serologic tests and CSF smear. Cell biopsy and nucleic acid amplification testing (traditional PCR, Xpert MTB (*Mycobacterium tuberculosis*)) were conducted according to clinically assessed necessity. Eligible patients were divided into six groups according to their final diagnoses: bacterial meningitis, tuberculous meningitis (TBM), viral encephalitis and/or meningitis, fungal meningitis, amoeba encephalitis and CNS non-infection. We included patients with definite or probable diagnoses of CNS bacterial meningitis [18], viral infections [19], TBM [20], and amoeba encephalitis. Fungal meningitis was confirmed by routine microbiological tests. Synchronous CSF samples were collected for mNGS. The inclusion criteria were as follows: (1) high-level clinical suspicion of CNS infectious disease; (2) CSF specimens submitted for mNGS testing. The exclusion criteria were as follows: (1) refusal to undergo lumbar puncture; (2) any contraindication for such puncture; (3) patients with unclear diagnosis at discharge by expert committee consultation.

### Sample sequencing and data analysis

CSF specimens of 1.5–3 mL were collected from each patient according to standard procedures. A 1.5 mL microcentrifuge tube containing 0.6 mL of sample, enzymes and a 1 g 0.5 mm glass bead were attached to a horizontal platform on a vortex mixer and agitated vigorously at 2800–3200 rpm for 30 min. Thereafter, DNA libraries were constructed through the process of DNA-fragmentation (approximately 150 bp fragments), end-repair, adapter-ligation, and PCR amplification. Agilent 2100 Bioanalyzer was used for quality control of the DNA libraries (200–300 bp). Quality-verified libraries were sequenced by the BGISEQ-50/MGISEQ-2000 platform [21]. High-quality sequencing data were generated by removing low-quality reads, followed by computational subtraction of human host sequences mapped to the human reference genome (hg19) using Burrows–Wheeler Alignment [22]. The remaining nonhuman sequences were read after subtraction of the human host sequences that were mapped to the human reference genome (hg19) using Burrows–Wheeler Alignment. The remaining data were compared with the microbial genome database (http://ftp.ncbi.nlm.nih.gov/genomes/), which includes 11, 910 bacteria, 7103 viruses, 1046 fungi, and 305 parasites related to human diseases. The mapped data were processed and analyzed, and the suspected pathogens were listed, including the number of reads, coverage, and depth of strict mapping. The sequencing result was shown in Additional file 1.

The criteria for evaluating microorganisms detected by mNGS were as follows:

(1) The microorganism detected by mNGS was consistent with the microorganism detected by conventional methods; (2) probable causative microorganisms were identified with reference to the literature and when the pathogenicity was consistent with the clinical manifestations [14].

### Data collection and diagnostic assessment of mNGS

Patients’ clinical data, including demographic characteristics, past medical history, immunosuppression status, laboratory examination, treatment process, and prognosis, were collected. Immunosuppression status included cases of progressive, solid, or haematological cancer, acquired immunodeficiency syndrome, administration of immunosuppressive drugs or steroids (at a dose greater than 0.3 mg/kg/day of prednisolone for at least 1 month) [23].

An expert group consisting of one microbiologist, one molecular biologist, and three infectious disease physicians adjudicated patients’ final clinical diagnoses according to the composite diagnostic criteria, after hospital discharge when the results of all the laboratory tests and patients’ responses to therapy were available [24]. The expert group members independently assessed each sample. When the results obtained were inconsistent, they reached a common conclusion through discussion.

The diagnostic performance of mNGS was assessed using the steps that follow. First, the patients were assigned to infected and non-infected groups. Second, the consistency of mNGS was compared with that of traditional methods and clinical diagnosis. Additionally, mNGS positive/case consistency indicated true-positive, while mNGS positive/case inconsistency indicated inconsistent results between mNGS results and final diagnosis. mNGS negative/case consistency indicated true-negative. mNGS negative/case inconsistency indicated missed detection of potential pathogen by mNGS when compared to the final diagnosis.

### Statistical analysis

Baseline characteristics and CSF laboratory indicators were statistically analysed, and quartiles were used to describe variables that did not conform to a normal distribution. Data were analysed by non-parametric and Fisher's exact tests, and p values < 0.05 were considered statistically significant. SPSS 23.0 software and GraphPad Prism 9 software were used for statistical analysis and data processing.

## Results

### General characteristics

Forty-seven patients were enrolled, of whom six were not included in the subsequent analysis due to unknown aetiology (Fig. 1). Of the remaining 41 patients, 31 were diagnosed with CNS infections and 10 were diagnosed with non-CNS infectious diseases. The most common aetiology was *Tubercular meningitis* (TB) infection, which accounted for 48.39% (15/31) of the cases. Seventeen patients were considered immunosuppressed. Common pathogens in immunosuppressed populations were MTB and other bacteria. Finally, CNS infections were categorised into MTB, other bacterial, fungal, viral, and amoebic infections. Non-CNS infections included autoimmune encephalitis, malignant tumours, and psychological disorders. There were no significant differences in baseline characteristics between the selected patients (Table 1).

**Table 1:** Baselines characteristics of participants

	CNS infection (n = 31)	Non-CNS infection (n = 10)	p value
Gender (n)			0.713
Male	23	8	
Female	8	2	
Age, year (range)	47.70 (4–75)	40.09 (5–73)	0.322
Body temperature max, ℃ (range)	39.00 (37.40–40.40)	38.30 (37.6–40.00)	0.084
Empirical treatment history (n)			0.433
Yes	30	9	
No	1	1	
Blood laboratory examination (range)			
WBC, × 10 9/L	7.44 (0.92–17.7)	5.55 (2.78–10.1)	0.316
Neutrophil, × 10 9/L	4.51 (0.53–15.18)	3.49 (1.25–8.28)	0.379
CRP, mg/L	9.80 (0–147.4)	6.75 (0.5–158.0)	0.761
PCT, ng/mL	0.097 (0.00–8.56)	0.059 (0.01–0.57)	0.129
CNS infection (n = 31)			
Bacterial infection	4	–	–
TB infection	15	–	–
Viral infection	9	–	–
Fungal infection	2	–	–
Amoeba infection	1	–	–
Non-CNS infection (n = 10)			
Malignant tumor	–	1	–
Hematological disease	–	2	–
Rheumatic disease	–	1	–
Psychological disease	–	2	–
Fever caused by infection	–	2	–
Other diseases	–	2	–

Fever caused by infection: fever caused by infection other than a CNS infection

### CSF inflammatory biomarker concentrations in different diagnostic groups

**Fig. 2:**
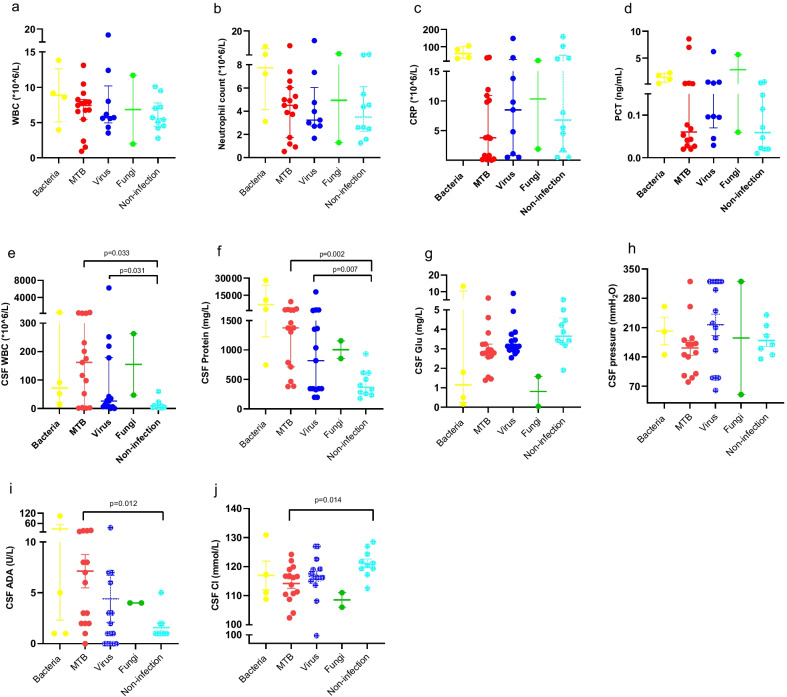
Inflammatory CSF and plasma biomarkers in different diagnostic groups. Scatter plots depicting levels of inflammatory plasma biomarkers: **a** WBC. **b** N. **c** CRP. **d** PCT. Scatter plots depicting levels of inflammatory CSF biomarkers: **e** CSF WBC. **f** CSF Protein. **g** CSF Glu. **h** CSF pressure. **i** CSF ADA. **j** CSF Cl. WBC: White blood cell; N: Neutrophile; CRP: C-reactive protein; PCT: Procalcitonin; ADA: Adenosine deaminase; Cl^−^: Chloride; Glu Glucose

Figure 2 displays CSF and plasma levels of the inflammatory markers in the different diagnostic groups. CSF white blood cell (WBC) count and protein levels were higher in patients with MTB and virus groups compared with the non-infection group (p = 0.033 and 0.031, p = 0.002 and 0.007, respectively). Furthermore, adenosine deaminase and chloride ion (Cl^−^) concentration of CSF were higher in patients in the MTB group compared with the non-infection group (p = 0.012 and 0.014, respectively). However, no obvious difference in plasma inflammatory biomarkers was found among different diagnostic groups.

### Overall diagnostic performance of mNGS

**Fig. 3:**
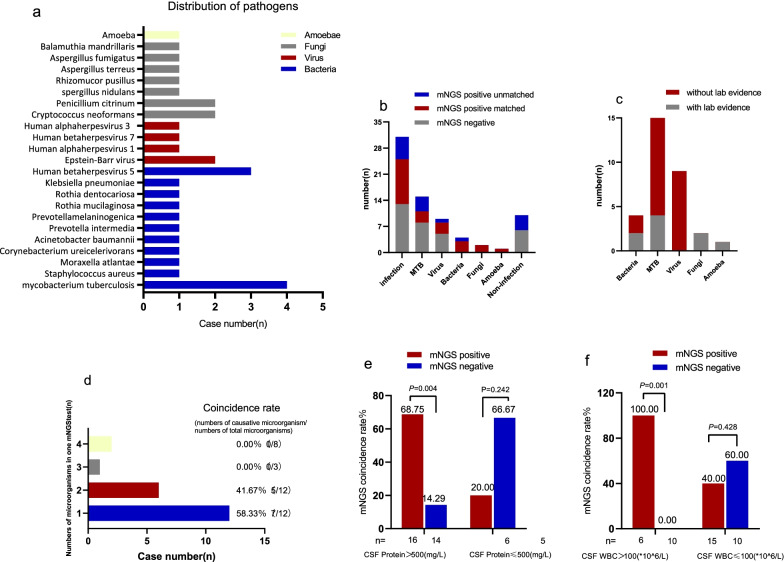
**a** Distribution of pathogens identified by mNGS. **b** Distribution of mNGS results in final diagnosis results. **c** The proportion of clinical and laboratory diagnosis in final diagnosis. **d** Number of pathogens detected in a single mNGS test. **e**, **f** The influence of protein and WBC in CSF on the coincidence rate between mNGS results and clinical diagnosis. Statistical methods (**e**, **f**): Fisher’s exact probability method

In total, 11 positive results were reported in the culture (including one false positive (dental actinomycetes) after evaluation), and mNGS detection revealed that MTB was the most commonly identified potential pathogen (n = 4), as shown in Fig. 3a. Cryptococcal meningitis was the only fungal infection observed in the present study. Seventeen non-pathogenic microorganisms were identified. As shown in Table 2, compared with traditional methods, the sensitivity and specificity of mNGS were 90.91% (9/10) and 63.33% (6/10).

**Table 2:** Comparison of sensitivity and specificity between mNGS, conventional methods, and clinical diagnosis

	Sensitivity	Specificity
mNGS/conventional methods	90.91% (10/11)	63.33% (19/30)
mNGS/clinical diagnosis	54.84% (17/31)	60.00% (6/10)
Conventional methods/clinical diagnosis	32.26% (10/31)	90.00% (9/10)

### Inconsistency analysis between mNGS and final clinical diagnosis

In 31 patients with CNS infections, the positivity rate of traditional methods was 32.26% (10/31), and that of mNGS was 54.84% (17/31). The coincidence rate of the former with the clinical diagnosis was 100% (10/10), and that of the latter was 70.59% (12/17). However, in 10 patients with non-CNS infection, mNGS showed 60.0% agreement with the final clinical diagnosis.

Among the enrolled 41 patients, the mNGS results of 12 patients were classified into the mNGS-positive/case-consistent group, while six patients’ mNGS results were categorised as mNGS-negative/case-consistent. mNGS failed to detect two MTB cases using traditional methods of positive CNS MTB infection. Nine cases were identified as mNGS-positive/case-inconsistent. Furthermore, mNGS did not detect the pathogen in 45.16% (14/31) of the patients with a final diagnosis of CNS infection. These cases were classified as false negative.

**Fig. 4:**
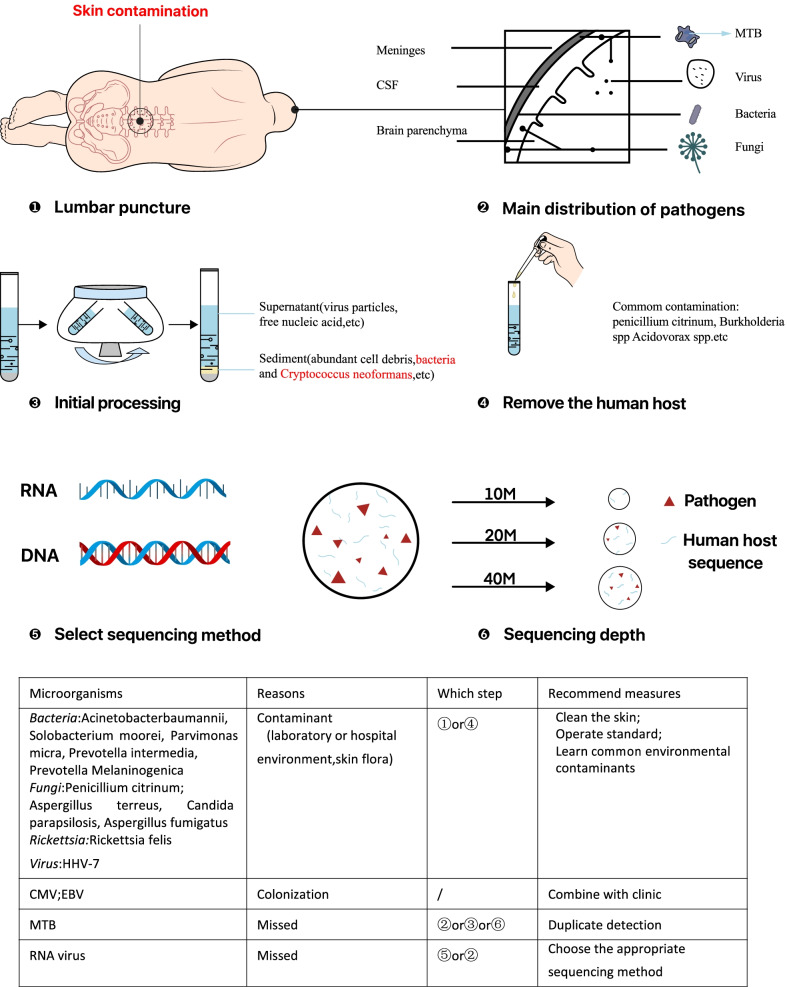
Simple flowchart of the mNGS process, highlighting common inconsistencies. ① Skin-derived microorganisms can be introduced during lumbar puncture, resulting in false positive mNGS results. ② The main distribution of different types of pathogens in the brain. ③ The supernatant was processed for further detection after centrifugation. ④ Common contaminants introduced when adding reagents for removing human hosts. ⑤ The choice of sequencing method also influenced the results. ⑥ Sequencing depth also affects the detection efficiency

Twenty-three cases did not conform to the final clinical diagnosis, accounting for more than half of the cases. In view of the above, the reasons for inconsistency were determined by the expert group with reference to relevant guidelines, consensus, and literature, combined with discussion and analysis of the patient’s clinical data. The reasons for this inconsistency are listed in Table 3 [14, 25–41]. More detailed information on results of routine biochemical and conventional methods have been submitted in Additional file 2. We summarized and sorted the above reasons, which are clearly marked in the intuitive mNGS process flow diagram (Fig. 4). Common inconsistencies, including common bacterial and fungal contaminants and frequent omissions, are summarized in the table in Fig. 4.

**Table 3:** The inconsistency analysis between mNGS results of CSF and clinical diagnosis in 23 patients

No	Immune function	Pathogen	mNGS results	Reads	Significance assessment	Basis for assessment
H210	Weak	Bacteria	*Solobacterium moorei*	6	Contaminant	1. The detected microorganisms are associated with oral infections [25, 26]. The patient had no history of dental surgery or chronic periodontitis [27, 28] 2. A rare report of central nervous system infection about detected microorganisms 3. The CSF was clear and odorless, staining was negative [29] 4. Four pathogens were detected at one test with few reads. It’s probably contamination
			*Parvimonas micra*	3	Contaminant	
			*Prevotella intermedia*	4	Contaminant	
			*Prevotella melaninogenica*	3	Contaminant	
H087	Weak	MTB	CMV	2172	Colonization	1. Detection of CMV in CSF in HIV does not imply CMV encephalitis [30] 2. CMV encephalitis often occurs in HIV with severely impaired immune function, with no specific manifestations and poor prognosis [31] 3. EBV is frequently detected in CSF of HIV patients, while only a few have primary CNS lymphoma [32] 4. Penicillium citrinum is common laboratory contaminant [33, 34] 5. No evidence of fungal histological infiltration. Symptoms improved without the anti-fungal drugs
			EBV	1772	Colonization	
			*Penicillium citrinum*	29	Contaminant	
			*Penicillium chrysogenum*	20	Contaminant	
S 273	Weak	MTB	*Rhizomucor pusillus*	7	Contaminant	1. No history of hematological tumors [35] 2. Common environmental pollutants, rarely causing infection [36] 3. No fungal infection manifestation, no histological evidence 4. Small number of sequences 5. Symptoms improved without the anti-fungal drugs
S 286	Normal	MTB	*Aspergillus terreus*	1	Contaminant	1. *Aspergillus terreus* were considered to be mNGS false-positive who did not have CNS aspergillosis [14] only 1 read. It’s probably contamination 2. Rickettsia Felis was 1 read; No myalgia or rash [37] 3. Non-infected was also detected in samples from healthy African populations [38]
			*Rickettsia felis*	1	Contaminant	
R004,005 H 049 H242 O284,285O288,297	Normal	MTB	Negative	/	False negative	1. MTB is an intracellular bacterium with low detection rate 2. CSF is a sterile body fluid with a low amount of MTB in an infected state 3. In the case of brain abscess, pathogen did not affect CSF currently 4. After centrifugation, MTB, cryptococcus ect are easy to be deposited below [39] 5. The amount of sequence at a time is 20 M
H088	Weak	Viral	*Moraxella atlantae*	149	Unclear	1. There are no reports of *Moraxella atlantae* and *Corynebacterium ureicelerivorans* infection with CNS so far, and the significance of both bacteria is unclear. Corynebacterium is generally considered a normal skin flora or contaminant. It is now often seen as an opportunistic pathogen in immunocompromised patients or those with severe conditions 2.Improved without anti-viral
			*Penicillium citrinum*	14	Contaminant	
			*Corynebacterium ureicelerivorans*	902	Unclear	
R021,K031H178, S267 J 277	Normal	Viral	Negative	/	False negative	1. RNA viruses probably. RNA viral encephalitis is also common [40, 41] 2. RNA are easily degraded 3. A low amount of virus pathogens 4. Some viruses exist mainly in cells
H 314	Weak					
R 013	Normal	Non-infection	*Candida parapsilosis*	9	Contaminant	1. Normal immune function, no susceptible factors 2. No evidence of fungal infection 3. Symptoms improved without antibiotics
H 094	HIV	Non-infection	*Acinetobacter baumannii*	5	Contaminant	1. No history of brain surgery 2. No symptoms of CNS infection 3. Few reads, it’s probably contamination
O291	Normal	Non-infection	HHV-7	7	Colonization	1. Acute encephalitis due to HHV-7 rarely occurs in immunocompetent adults [42] 2. No symptoms of CNSH HV-7 infection 4. Improved without anti-viral
H 311	Weak	Non-infection	*Aspergillus fumigatus*	2	Contaminant	*Aspergillus fumigatus* was considered to be mNGS false-positive who did not have CNS aspergillosis. only 2 reads

*mNGS* metagenomic next-generation sequencing, *MIS* Meningeal irritation sign, *CSF* cerebrospinal fluid

In our study, eight mNGS results were evaluated as contamination, and based on clinical conditions, six of them were found to be laboratory fungal contamination. These microorganisms were not within the scope of common skin pollutants and were therefore considered laboratory contaminants. Thirteen mNGS results were evaluated as false negatives, including eight MTB and five viral infections. The main reasons for the omission of MTB or viral infection are shown in Fig. 4 (MTB—②, ③, ⑥, and virus—②, ⑤). Cytomegalovirus (CMV) and Epstein–Barr virus (EBV) colonisations were detected by mNGS in three patients, two of which were immunosuppressed.

### Correlative analysis between mNGS and CSF laboratory results

In patients with CSF WBC > 100^10^6^/L and CSF protein > 500 mg/L, the coincidence rate of the mNGS-positive group was significantly higher than that of the mNGS-negative group (100.00% vs. 0.00%, p < 0.001; 68.75% vs. 14.29%, p = 0.004). In patients with CSF WBC count < 100^10^6^/L and CSF protein level < 500 mg/L, the coincidence rate of the mNGS-negative group was higher than that of the mNGS-positive group (60.00% vs. 40.00%, p = 0.428; 66.67% vs. 20.00%, p = 0.242). However, there was no statistically significant difference in the coincidence rate between the two groups (Fig. 3d, e).

### Results obtained from the evaluation of mNGS according to the model we explored

**Fig. 5:**
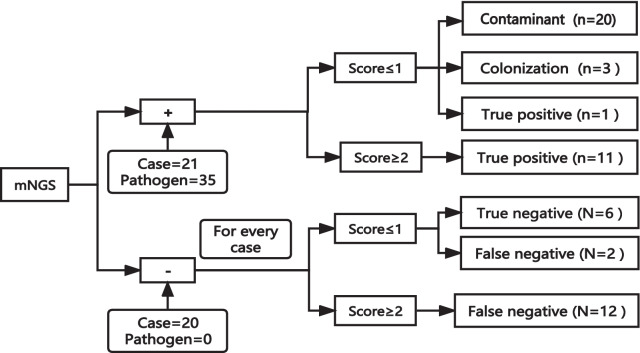
Evaluation process of mNGS results. mNGS+: mNGS positive; mNGS−: mNGS negative; n: number of pathogens; N: number of cases

Given our final diagnosis, a model for evaluating the mNGS results was explored, and the results are shown in Fig. 5. For each pathogen detected by mNGS, each influencing factor (including typical clinical features, traditional positive method, and CSF cells ≥ 100 (10^6/L) or protein ≥ 500 mg/L; one point for each item) was scored according to the actual situation; the points were then added together to obtain the total score. Most pathogens were regarded as contamination when the final score was ≤ 1. The mNGS results were true positives, consistent with the clinical diagnosis when the score was ≥ 2. For negative mNGS results, each case was evaluated using the aforementioned scoring system.

## Discussion

We assessed the diagnostic performance of mNGS and conventional methods in comparison with final clinical diagnosis. More importantly, we analysed the reasons for the inconsistency between mNGS results and the final clinical diagnosis and provided a reasonable explanation. Based on the process of case analysis, a phenomenon, which attempts to provide a reference for clinicians to analyse the mNGS results of suspected CNS infections, was noted, given the current lack of a standard evaluation process for mNGS results. The overall consistency rate of mNGS was 43.90%, including 12 positive and six negative mNGS results. We obtained similar results to those reported in previous studies [14, 24, 42]. These findings show that inconsistency between mNGS results and clinical diagnosis is common, and thus mNGS results must be carefully interpreted in combination with the patient’s clinical situation.

The overall percentage of study patients diagnosed with CNS infection (75.61% [31 patients]) was higher than the 29–60% reported in the literature [5, 6, 43]. Most CSF samples of the 41 patients for mNGS testing were obtained after the patients were exposed to empirical antibiotics, thus potentially decreasing the diagnostic yield. Moreover, there was no significant difference in plasma inflammation biomarkers in the different diagnostic groups, which probably biased the enrolment of patients who were particularly difficult to diagnose using conventional methods.

Of the 19 infections missed by mNGS, 17 were diagnosed by comprehensive evaluation based on clinical manifestations, since they lacked aetiological evidence. It is worth noting that the retrospective results indicated that mNGS failed to identify 11 out of 15 TB meningitis cases, with a detection rate of only 26.67%, which is lower than the reported rate of 27.3–84.44% [5, 6, 43]. Similar to PCR and antigen-based tests, mNGS is a direct test that relies on the presence of pathogenic nucleic acids in samples. We speculated that this was mainly due to the low abundance of MTB, effects of empirical antibiotics, and sequencing depth [14]. MTB was detected by mNGS four times with reads of 1–18; these four cases were diagnosed as tuberculous meningitis (TBM) by a comprehensive assessment. Due to the low possibility of contamination, mNGS has strong implications for TBM diagnosis when at least one specific read is matched to the MTB complex, which is consistent with the findings of previous studies [16, 17]. Although mNGS was highly sensitive in identifying a pathogenic causative organism in CSF samples, our findings indicate that a negative mNGS result should be interpreted with caution owing to the higher risk of false-negative results. Timely diagnostic antituberculosis treatment for patients suspected of having TBM is crucial for prognosis, as it is a fatal form of tuberculosis infection [20]. Many cases have been treated without evidence of the pathogen [7, 17, 45].

Among the nine patients with CNS viral infection (five were negative for mNGS), herpes simplex virus (HSV-1) and varicella zoster virus (VZV) detected by mNGS were common DNA viruses of viral encephalitis, consistent with the findings of other studies [20, 45]. The efficiency of mNGS in diagnosing viral encephalitis and/or meningitis is not satisfactory, possibly attributable in part, to the absence of mNGS RNA detection. RNA viruses, such as enteroviruses and Japanese encephalitis viruses, are common causes of viral encephalitis and meningitis [40, 41]. Therefore, we suggest that patients who was suspected of CNS viral infections in clinically undergo routine biochemical testing of CSF and PCR testing of common encephalitis viruses (such as HSV, VZV, and CMV) before antibiotic treatment and store 2 ml CSF in a − 80 °C refrigerator simultaneously, when the pathogen was not identified after these detections and the symptoms do not relieve after 3 days, the stored CSF will be submitted for mNGS RNA testing to maximize its value [46]. The influence of empirical antibiotics on the mNGS results remains controversial. However, some studies have shown that antibiotics have little influence on mNGS results. It is difficult to evaluate the influence of antibiotics on mNGS results in our study because most patients were treated empirically prior to CSF sampling.

Contamination may occur during sampling and laboratory operations, as shown in Fig. 4, introducing microorganisms unrelated to the cause of the disease, resulting in false-positive results. Additionally, the type and cellularity of the pathogen can affect the detection rate. Furthermore, sample processing and the absence of RNA detection may lead to negative mNGS results. However, the effects of antibiotics cannot be disregarded.

Furthermore, EBV DNA was detected in two patients, but there was no evidence of primary CNS lymphoma on magnetic resonance imaging, and the two patients responded well without anti-EBV treatment. Similarly, CMV DNA was identified in two patients. EBV and CMV in the CSF of the CNS are common in patients with acquired immunodeficiency syndrome, but the incidence of primary CNS lymphoma or cytomegalovirus encephalitis is low [30–32]. We knew from the aforementioned results that colonisation is another reason for false-positive results, especially in immunosuppressed patients.

As mentioned above, inconsistencies between mNGS results and clinical diagnoses are common. False-positive results are likely to mislead therapeutic decision-making and false-negative results can leave clinicians at a loss. Therefore, a reasonable interpretation of mNGS results is particularly important. A practical method to evaluate the results of mNGS has yet to be established.

We explored an integral method to evaluate mNGS, given our final diagnosis, to provide a reference for clinicians to analyse mNGS results for suspected CNS infection, as shown in Fig. 5. Each pathogen detected by mNGS was scored independently, according to the scoring system described above. Each case was scored. From the results, it is evident that a negative mNGS result does not necessarily mean that the patient was not infected clinically. Our results showed that regardless of whether the mNGS result was positive or negative, the possibility of infection was greater when the score was ≥ 2. For all laboratory tests, mNGS results must be interpreted in conjunction with clinical data, preferably in a multidisciplinary manner [47].

This study had several limitations. First, it was a single-centre retrospective study with a small sample size. Second, almost all patients were treated empirically before CSF sampling, and the effect of antibiotics on mNGS detection rates in this retrospective analysis was unknown. Finally, clinical diagnosis was used as the gold standard in this study, of which more than half was not confirmed by aetiological evidence.

## Conclusions

Inconsistencies between mNGS results and clinical diagnoses are common. mNGS results need to be comprehensively analysed in combination with clinical symptoms of patients, pathogenicity, cell and protein levels in CSF, reads of microorganisms, and host immune status. The integral method is effective for evaluating mNGS results. Regardless of whether the mNGS result was positive or negative, the possibility of infection was greater when the score was ≥ 2. A negative mNGS result does not necessarily indicate that the patient was not clinically infected, and, therefore, clinical features are more important.

## Supplementary Information

**Additional file 1:** The results of next generation sequencing of cerebrospinal fluid.

**Additional file 2:** Comparison of the etiological results between conventional detection methods and next-generation sequencing technology.

## Data Availability

The datasets analysed during the current study are available in the GenBank of National Center for Biotechnology Information (NCBI) repository, which can be accessible with the following link: https://www.ncbi.nlm.nih.gov/sra/PRJNA871996.

## Abbreviations

CNS: Central nervous system
CSF: Cerebrospinal fluid
CMV: Cytomegalovirus
EBV: Epstein–Barr virus
mNGS: Metagenomic next-generation sequencing
MTB: Mycobacterium tuberculosis infection
PCR: Polymerase chain reaction
WBC: White blood cell
TBM: Tuberculous meningitis
